# Supplementary material for: The recombination landscape of the Khoe-San likely represents the upper limits of recombination divergence in humans
Source: Genome Biol. 2022 Aug 9;23:172. doi: 10.1186/s13059-022-02744-5 (PMC9361568; doi:10.1186/s13059-022-02744-5)
Supplement: Supplementary file 1 — Additional file 1: Figure S1. Ancestry estimates for the Nama obtained using ADMIXTURE with k=4 to k=7 possible ancestral clusters. Figure S2. (A) Ternary diagram showing the African, European and Khoe-San ancestry contributions, as represented by the LWK, GBR and Nama respectively, for the whole WGS dataset of 84 individuals. (B) Ternary diagram showing the African, European and Khoe-San ancestry contributions, as represented by the LWK, GBR and Nama respectively, for the unrelated subset of the WGS dataset. [file 13059_2022_2744_MOESM1_ESM.pdf]

Supplementary Figures - The recombination landscape of the Khoe-San likely represents the upper limits of recombination divergence in humans

Gerald van Eeden[1], Caitlin Uren[1,2], Evlyn Pless[3], Mira Mastoras[3], Gian D. van der Spuy[1,2,4], Gerard Tromp[1,2,4], Brenna M. Henn[3*], Marlo Möller[1,2*]

[1]DSI-NRF Centre of Excellence for Biomedical Tuberculosis Research, South African Medical Research Council Centre for Tuberculosis Research, Division of Molecular Biology and Human Genetics, Faculty of Medicine and Health Sciences, Stellenbosch University, Cape Town, South Africa

[2]Centre for Bioinformatics and Computational Biology, Stellenbosch University, 7602 Stellenbosch, South Africa

[3]Department of Anthropology, Center for Population Biology and the Genome Center, University of California (UC) Davis, Davis, CA, USA

[4]SAMRC-SHIP South African Tuberculosis Bioinformatics Initiative (SATBBI), Center for Bioinformatics and Computational Biology, South Afric

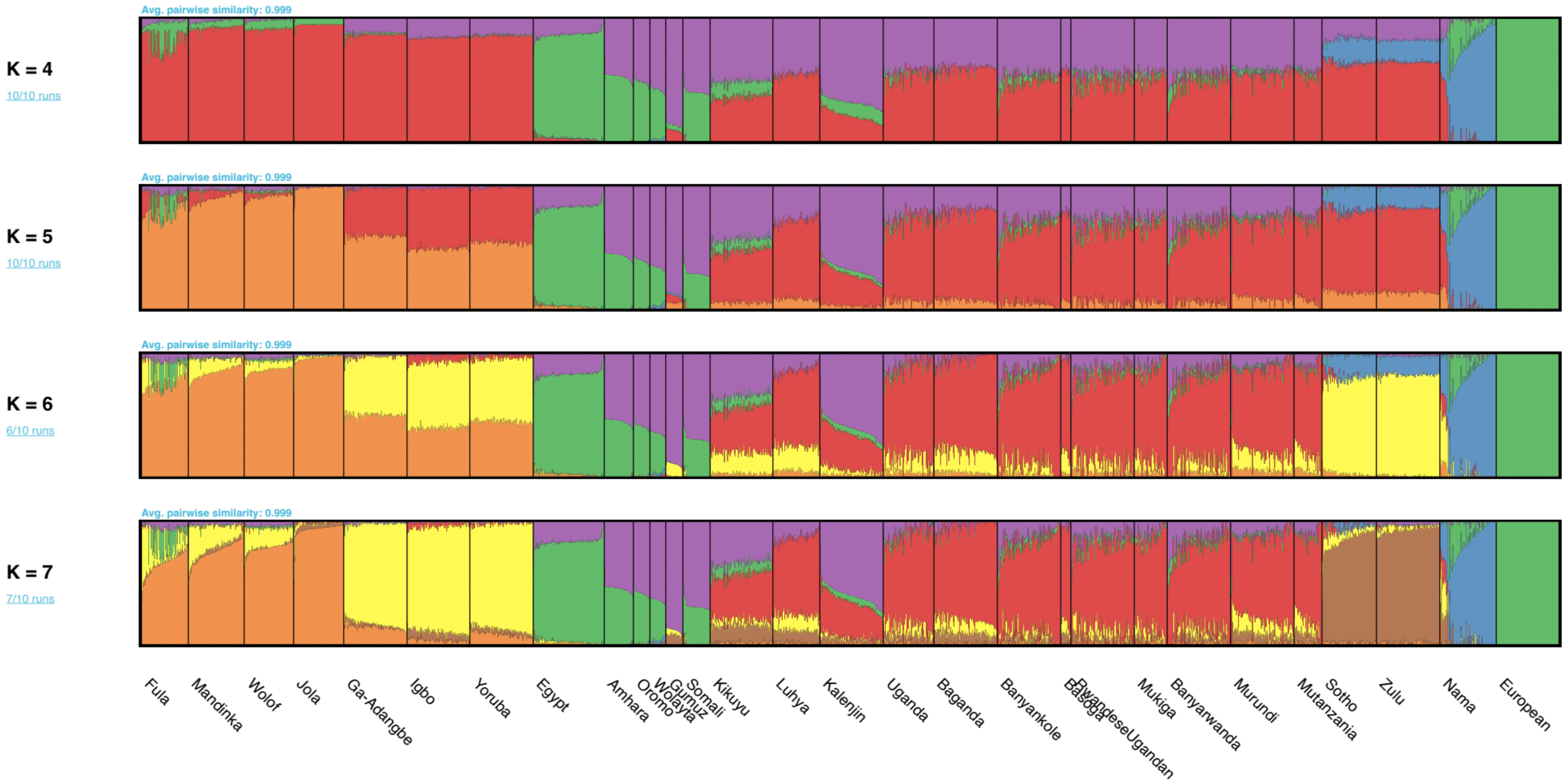

Supplementary Figure S1: Ancestry estimates for the Nama obtained using ADMIXTURE with k=4 to k=7 possible ancestral clusters.

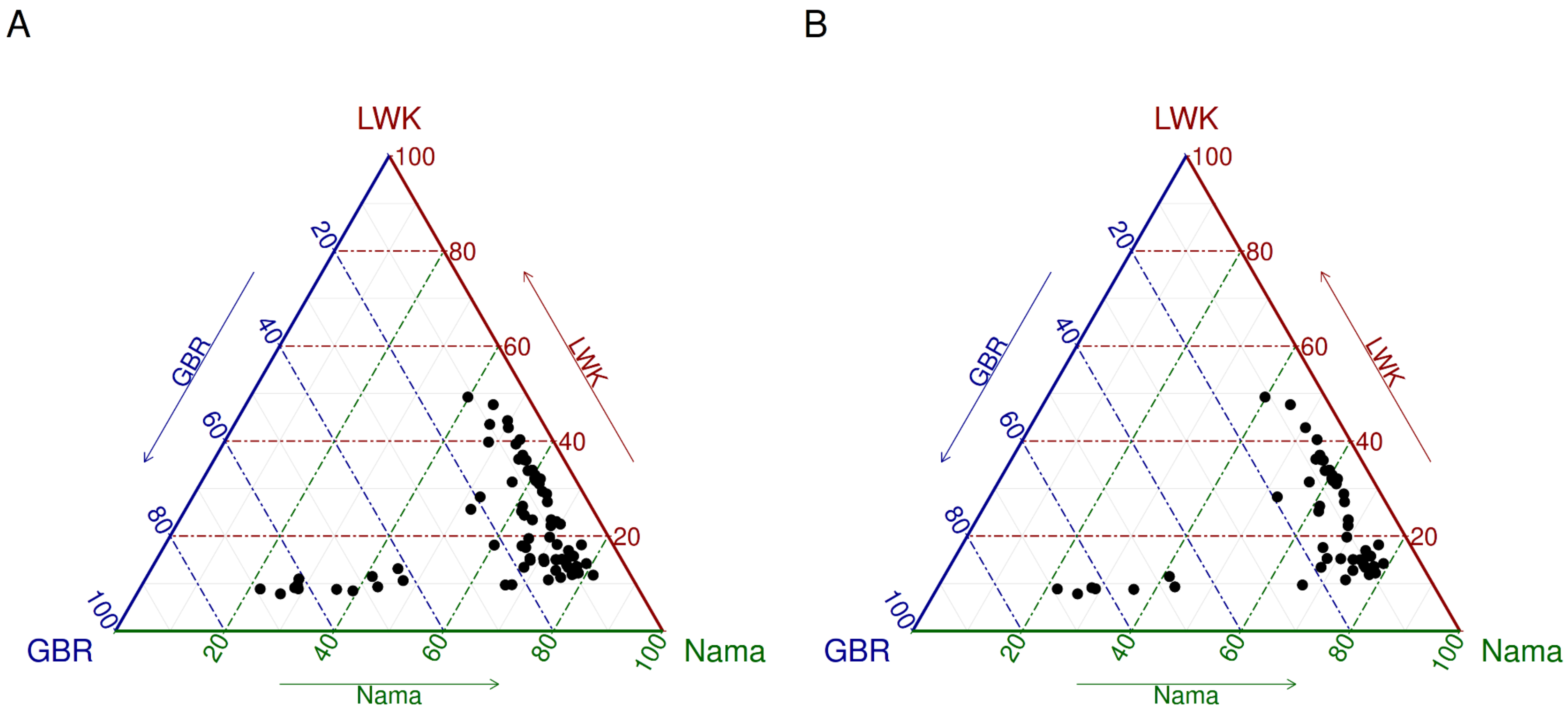

Supplementary Figure S2: (A) Ternary diagram showing the African, European and KhoeSan ancestry contributions, as represented by the LWK, GBR and Nama respectively, for the whole WGS dataset of 84 individuals. (B) Ternary diagram showing the African, European and KhoeSan ancestry contributions, as represented by the LWK, GBR and Nama respectively, for the unrelated subset of the WGS dataset.
