# Supplementary material for: The recombination landscape of the Khoe-San likely represents the upper limits of recombination divergence in humans
Source: Genome Biol. 2022 Aug 9;23:172. doi: 10.1186/s13059-022-02744-5 (PMC9361568; doi:10.1186/s13059-022-02744-5)
Supplement: Supplementary file 2 — Additional file 2. [file 13059_2022_2744_MOESM2_ESM.docx]

**Review history**

**First round of review**

**Reviewer 1**

In this paper van Eden and colleagues infer a demographic history and a population-specific fine-scale recombination map for the Nama, a Khoe-San population in southern Africa. The authors then compare this recombination map to those estimated using the same method in all of the One Thousand Genomes populations and find that it is substantially different from any other recombination map. Finally, the authors show that this has implications for selection scans using iHS. Overall I think that the paper is well-written, the methods are straightforward but carefully done, and the resulting recombination map will be of interest and use to the broader community. I have a couple of minor comments about the interpretation that I list below.


Minor Comments:

- The authors should note some caution in their interpretation of Figure 2. There are two distinct components that make up the correlation of the inferred genetic maps of two populations -- first, there is the correlation of the true (but unobserved) genetic map, and second, there is the noise in going from the true genetic map to the inferred genetic map. As an example, the true Nama recombination map might be identical to the true LWK recombination map, but if it is inferred with a lot of noise the inferred recombination map will have a low correlation with LWK (and with all other populations). The sample size in the Nama is a bit lower than that for the populations in the 1KG and given the recent bottleneck in the Nama, there may be even more noise for inferring recombination maps for the Nama even at similar sample sizes. I find it plausible (likely even) that the recombination map of the Nama is substantially different from other populations, but the authors may want to highlight that the degree to which this is true is lower bounded by the results in Figure 2 (i.e., the true Nama recombination map is likely to be more similar to other recombination maps than suggested by Figure 2). It would be outside of the scope of the present manuscript, and not necessary for revision, but if interested, the authors could get at this question by estimating the noise in the estimated recombination maps via simulations (e.g., simulating data for two populations under a joint demographic history assuming that the population have the same genetic map and then seeing how correlated the resulting inferred maps are).

- Given the amount of gene flow between the Nama and other populations (represented here by LWK and GBR) the authors may want to acknowledge the limitations of LD-based methods in inferring recombination rates (see e.g., Samuk and Noor "Gene flow biases population genetic inference of recombination rate" https://www.biorxiv.org/content/10.1101/2021.09.26.461846v1). It seems like what happens is that in the presence of gene flow the resulting recombination maps are something of a mixture of the recombination maps present in the the different component populations. This is not a serious issue for the present work, but something the authors may want to make note of. Another suggestion (which is again outside the score of the present manuscript and not necessary for revision) would be to mark all of the regions that RFmix identifies as being from GBR or LWK in an individual as missing, and run pyrho on this masked dataset. That way only individuals with putative Nama ancestry in a given window are used to estimate the recombination rate in that window. On one hand this might more faithfully represent the historical recombination rate in the Nama, but on the other hand it is not obvious to me whether it is better for a given downstream application for a recombination map to accurately estimate the recombination map in the "primary ancestry" of an admixed population, or to accurately estimate a sort of "ancestry-averaged" recombination map.

**Reviewer 2**

In the proposed manuscript, van Eeden and colleagues have presented a recombination map for southern Africa "Khoe-San" Nama population. The ancestors of Khoe-San groups form the first population divergence split - between Khoe-San and all other living populations. Consequently, currently available recombination map can be potentially problematic when studying the genetic variation of native southern Africa groups. The proposed study is well written and thought out and it seem to follow the authors previous study van Eeden et al. (2021). Given the data availability, the authors argumentation for using LD-based methods to estimate the recombination map seems valid and Ne estimations using SMC++ and IBDNe algorithms is adequate. I can only think of minor comments:
- During the selection scans of 104 Nama individuals, the authors have excluded ~48% of the dataset due to admixture and relatedness. Given the potential heterogeneity of genetic ancestries between the 104 Nama individuals dataset, it made me wonder how heterogeneous is the sampling from which the recombination map is based on. Some supplementary figures could help the reader understand the genetic variation of the studied population.
- Effective population size (Ne) should be in italic. Eg. Page 5 line98, 109, 110
- Some citations seem not to be properly formatted during the text: Voight 2006, Zhang 2012

**Authors’ response to reviewers**

**Reviewer #1:**

In this paper van Eden and colleagues infer a demographic history and a population-specific fine-scale recombination map for the Nama, a Khoe-San population in southern Africa. The authors then compare this recombination map to those estimated using the same method in all of the One Thousand Genomes populations and find that it is substantially different from any other recombination map. Finally, the authors show that this has implications for selection scans using iHS. Overall I think that the paper is well-written, the methods are straightforward but carefully done, and the resulting recombination map will be of interest and use to the broader community. I have a couple of minor comments about the interpretation that I list below.

Minor Comments:

## - The authors should note some caution in their interpretation of Figure 2. There are two distinct components that make up the correlation of the inferred genetic maps of two populations -- first, there is the correlation of the true (but unobserved) genetic map, and second, there is the noise in going from the true genetic map to the inferred genetic map. As an example, the true Nama recombination map might be identical to the true LWK recombination map, but if it is inferred with a lot of noise the inferred recombination map will have a low correlation with LWK (and with all other populations). The sample size in the Nama is a bit lower than that for the populations in the 1KG and given the recent bottleneck in the Nama, there may be even more noise for inferring recombination maps for the Nama even at similar sample sizes. I find it plausible (likely even) that the recombination map of the Nama is substantially different from other populations, but the authors may want to highlight that the degree to which this is true is lower bounded by the results in Figure 2 (i.e., the true Nama recombination map is likely to be more similar to other recombination maps than suggested by Figure 2). It would be outside of the scope of the present manuscript, and not necessary for revision, but if interested, the

**authors could get at this question by estimating the noise in the estimated recombination maps via simulations (e.g., simulating data for two populations under a joint demographic history assuming that the population have the same genetic map and then seeing how correlated the resulting inferred maps are).**

Thank you for this suggestion. We agree and have included the following disclaimer where the relevant results are discussed at the top of page 8:

Furthermore, we find that the Nama are more closely related to other African populations than to other continental groups (< 0.75), however, the pairwise correlations between the Nama and the other African populations are much weaker (~0.79) than the pairwise correlations between the African populations (> 0.90). These values represent correlations between inferred maps and, therefore, include any noise potentially introduced during inference. The true maps are likely to be more similar than these values suggest.

**- Given the amount of gene flow between the Nama and other populations (represented here by LWK and GBR) the authors may want to acknowledge the limitations of LD-based methods in inferring recombination rates (see e.g., Samuk and Noor "Gene flow biases population genetic inference of recombination rate"** [**https://www.biorxiv.org/content/10.1101/2021.09.26.461846v1**](https://www.biorxiv.org/content/10.1101/2021.09.26.461846v1)**). It seems like what happens is that in the presence of gene flow the resulting recombination maps are something of a mixture of the recombination maps present in the different component populations. This is not a serious issue for the present work, but something the authors may want to make note of.**

Thank you for this suggestion. We agree that this is a relevant factor to make known to readers. We added the following when discussing assumptions of LD-based methods at the bottom of page 11:

Our small sample size (54 unrelated individuals) made LD-based methods the obvious choice for fine-scale estimates. However, there are many assumptions that accompany LD-based methods that make them less than ideal, for instance the assumption of a constant Ne and the potential bias from gene flow when inferring recombination in admixed populations.

## Another suggestion (which is again outside the score of the present manuscript and not necessary for revision) would be to mark all of the regions that RFmix identifies as being from GBR or LWK in an individual as missing, and run pyrho on this masked dataset. That way only individuals with putative Nama ancestry in a given window are used to estimate the recombination rate in that window. On one hand this might more faithfully represent the historical recombination rate in the Nama, but on the other hand it is not obvious to me whether it is better for a given downstream application for a recombination map to accurately estimate the recombination map in the "primary ancestry" of an admixed population, or to accurately estimate a sort of "ancestry-averaged" recombination map.

Thank you for this suggestion. This is something we considered, however as you mention, we wanted the resultant map to be used for other downstream applications. Therefore, we wanted a map that is representative of the current population. The averaging effect from including segments from other ancestries is something to be aware of when using the map for downstream applications though. It might be prudent to create a map solely from the Nama component for certain recombination specific studies or for selection studies. However, there are also sample size considerations when pursuing this. The depth of representative Nama segments across the genome in our dataset likely varies considerably and would affect the accuracy of the resultant map across the genome.

**Reviewer #2:**

## In the proposed manuscript, van Eeden and colleagues have presented a recombination map for southern Africa "Khoe-San" Nama population. The ancestors of Khoe-San groups form the first population divergence split - between Khoe-San and all other living populations. Consequently, currently available recombination map can be potentially problematic when studying the genetic variation of native southern Africa groups. The proposed study is well written and thought out and it seem to follow the authors previous study van Eeden et al. (2021). Given the data availability, the authors argumentation for using LD-based methods to estimate the recombination map seems valid and Ne estimations using SMC++ and IBDNe algorithms is adequate. I can only think of minor comments:

## During the selection scans of 104 Nama individuals, the authors have excluded ~48% of the dataset due to admixture and relatedness. Given the potential heterogeneity of genetic ancestries between the 104 Nama individuals dataset, it made me wonder how heterogeneous is the sampling from which the recombination map is based on. Some supplementary figures could help the reader understand the genetic variation of the studied population.

Thank you for this suggestion. The Nama recombination map is based on 84, not 104, individuals. The 104 individuals is the SNP array dataset (encompassing the 84 sequenced individuals) used in a selection scan. Even so, we agree with the suggestion to include figures on the genetic variation of the studied population. We have included admixture plots and ternary diagrams as supplementary figures to visualise the proportion of the three primary ancestries in the Nama, as utilised in the ancestry-specific IBDNe inference.

## Effective population size (Ne) should be in italic. Eg. Page 5 line98, 109, 110

Thank you, we have made the necessary amendments throughout the article.

## Some citations seem not to be properly formatted during the text: Voight 2006, Zhang 2012

Thank you for noticing this, we have made the necessary amendments throughout the article.

**New additions:**

We have included the following section at the start of the Results section to better describe the how the WGS data were generated and elaborate on the expected ancestry proportions in the Nama:

Briefly, 84 Nama individuals were sequenced to 4x-8x depth via Illumina short read sequencing, variant-called and phased in combination with additional African low coverage genomes as well as 1000 Genomes Phase 3 as part of the African Genome Resource [[30]](https://sciwheel.com/work/citation?ids=724260&pre&suf&sa=0). Genomes were variant-called with GATK3.4 following best practices and phased with SHAPEIT2. Further details regarding the production of this dataset are described in Ragsdale et al. [[27]](https://sciwheel.com/work/citation?ids=13298170&pre&suf&sa=1). Global ancestry estimates for the Nama, as compared to other Africans from the African Genome Resource along with representative Europeans (CEU), were inferred using ADMIXTURE. Ancestry estimates indicate that the bulk of the Nama’s ancestry is Khoe-San, which is rare elsewhere in the African continent with the exception of the southern Bantu-speaking Sotho and Zulu (Figure S1). There is a sharp cline in European ancestry across individuals, ranging from ~0-50% as may occur with a recent pulse of admixture which has not yet reached equilibrium in a few generations. A subset of individuals carry ancestry frequent in Bantu-speaking and eastern African populations, likely reflecting recent Damara or Herero marriage as indicated in demographic interviews with participants. Ancestry proportion among the full set of related individuals was similar to the subset of unrelated individuals (Figure S2).

We have also included the following section to better describe our reasoning for using SNP array data of comment on the LAI accuracy:

We tested the accuracy of RFMix via simulation in the Nama as well as testing both SNP array and low coverage genome data in order to determine the best dataset for local ancestry inference. We simulated continuous gene flow from 3 ancestral groups: European admixture starting 8 generations ago with 1% contribution per generation, Bantu admixture starting 14 generations ago with 2% contribution per generation, and the remaining contribution for each generation coming from the KhoeSan. The population randomly mates to create each subsequent generation, also taking into account the recombination landscape to accurately copy haplotype blocks. Eleven individuals were used for each ancestral population: French individuals as the European reference, Bantu-speaking as the West African reference, and Nama individuals with >90% KhoeSan ancestry (that were not later used in RFmix runs) for the KhoeSan ancestry component. The average global LAI accuracy, allowing the reference individuals to themselves be admixed, was ~92% on average for the simulated individuals. European ancestry-specific accuracy was 97.6% with individuals being 12.8% European on average, Khoe-San ancestry-specific accuracy was 92.4% with individuals being 76.8% KhoeSan, and Bantu accuracy was 81.5%, with individuals having 10.4% Bantu ancestry overall.

Comparing RFmix runs for the SNP array and genome data to previously obtained ADMIXTURE ancestry percentage estimates, we found that the KhoeSan ancestry was systematically under-called in the genomes compared to the global ancestry estimates from ADMIXTURE, with European and Bantu ancestry consistently higher. This trend is however improved when using the SNP-array data for LAI. Therefore in the AS-IBDNe analysis, admixture deconvolution was performed on MEGA SNP array [[33]](https://sciwheel.com/work/citation?ids=4553609&pre&suf&sa=0) data in order to facilitate larger numbers of haplotypes in the reference populations.
